# Metabolites Analysis of Anti-Myocardial Ischemia Active Components of *Saussurea involucrata* Based on Gut Microbiota—Drug Interaction

**DOI:** 10.3390/ijms23137457

**Published:** 2022-07-05

**Authors:** Hang Yu, Jie Fu, Hui-Hui Guo, Li-Bin Pan, Hui Xu, Zheng-Wei Zhang, Jia-Chun Hu, Xin-Yu Yang, Hao-Jian Zhang, Meng-Meng Bu, Yuan Lin, Jian-Dong Jiang, Yan Wang

**Affiliations:** State Key Laboratory of Bioactive Substance and Function of Natural Medicines, Institute of Materia Medica, Chinese Academy of Medical Sciences/Peking Union Medical College, Beijing 100050, China; yuhang@imm.ac.cn (H.Y.); fujie@imm.ac.cn (J.F.); guohh@imm.ac.cn (H.-H.G.); panlibin@imm.ac.cn (L.-B.P.); xuhui@imm.ac.cn (H.X.); zhangzhengwei@imm.ac.cn (Z.-W.Z.); hujiachun@imm.ac.cn (J.-C.H.); xinyuy20@mails.jlu.edu.cn (X.-Y.Y.); zhanghaojian@imb.pumc.edu.cn (H.-J.Z.); bumengmeng@imm.ac.cn (M.-M.B.); linyuan@imm.ac.cn (Y.L.)

**Keywords:** *Saussurea involucrata*, gut microbiota, *Tian-Shan-Xue-Lian*, LC-MS/MS, LC/MS^n^-IT-TOF, metabolite

## Abstract

*Saussurea involucrata* has been reported to have potential therapeutic effects against myocardial ischemia. The pharmacological effects of oral natural medicines may be influenced by the participation of gut microbiota. In this study, we aimed to investigate the bidirectional regulation of gut microbiota and the main components of *Saussurea involucrata*. We first established a quantitative method for the four main components (chlorogenic acid, syringin, acanthoside B, rutin) which were chosen by fingerprint using liquid chromatography tandem mass spectrometry (LC-MS/MS), and found that gut microbiota has a strong metabolic effect on them. Meanwhile, we identified five major rat gut microbiota metabolites (M1–M5) using liquid chromatography tandem time-of-flight mass spectrometry (LC/MS^n^-IT-TOF). The metabolic properties of metabolites in vitro were preliminarily elucidated by LC-MS/MS for the first time. These five metabolites of *Saussurea involucrata* may all have potential contributions to the treatment of myocardial ischemia. Furthermore, the four main components (10 μg/mL) can significantly stimulate intestinal bacteria to produce short chain fatty acids in vitro, respectively, which can further contribute to the effect in myocardial ischemia. In this study, the therapeutic effect against myocardial ischemia of *Saussurea involucrata* was first reported to be related to the intestinal flora, which can be useful in understanding the effective substances of *Saussurea involucrata*.

## 1. Introduction

Myocardial ischemia refers to a pathological state in which the blood perfusion of the heart is reduced, resulting in reduced oxygen supply to the heart and abnormal myocardial energy metabolism, so the body cannot support the normal work of the heart [1]. Myocardial ischemia, which can lead to heart failure, has affected nearly 26 million people worldwide [1]. At the same time, subsequent myocardial ischemia can induce ischemia-reperfusion injury, which often aggravates the symptoms of ischemic injury due to mitochondrial damage and oxidative stress [2]. It has been reported that decreased blood pressure, decreased aortic blood supply, coronary artery occlusion, atherosclerosis, and inflammation may induce myocardial ischemia [3].

Saussureae Involucratae Herba, *Tian-Shan-Xue-Lian*, is the dry aerial part of *Saussurea involucrata* of the Compositae family. Traditional Chinese medicine teaches that *Saussurea involucrata* has the effects of warming the kidney and helping the *yang*, dispelling wind and dampness, clearing the meridian and promoting blood circulation, and has a unique therapeutic effect on patients with myocardial ischemia [4]. Modern medicine has further explained the pharmacological activity of *Saussurea involucrata*, and found that *Saussurea involucrata* has many unique pharmacological activities such as myocardial protection, vascular regulation, anti-tumor, anti-inflammatory, and anti-rheumatic, scavenging free radicals, anti-fatigue, and immune regulation effects [5,6,7,8,9,10]. Notably, *Saussurea involucrata* has been reported in recent years to have therapeutic effect against myocardial ischemia by affecting Acetylcholine-activated K^+^ current [11], regulating lipid peroxidation activation and infracts size [12], and altering cyclic guanosine monophosphate (cGMP), inducible nitric oxide synthase (iNOS), and 3-nitrotyrosine concentrations [13], which correspond to the traditional Chinese medicine effect of *Saussurea involucrata* for clearing the meridian and promoting blood circulation. Studies have shown that *Saussurea involucrata* contains a variety of active ingredients with different chemical structures, such as flavonoids, lignans, phenolics, phenylpropanoids, and sesquiterpenes. Many of these components have been reported to have pharmacologic activity against myocardial ischemia [11,13,14], which are often affected by the interaction between the body and the drug.

Gut microbiota refers to various types of bacteria, fungi, and bacteriophages that exist in the intestinal tract of the body. According to reports, there are more than 1000 kinds of bacteria in the gut, which are rich in many enzymes related to material metabolism [15], and some researchers have proposed to use the gut microbiota as “the second metabolic organ other than the liver” [16]. Therefore, the gut microbiota plays an important role in the biotransformation and absorption of oral drugs. Different from hepatic metabolism, the types of biotransformation caused by gut microbiota are unique, and the metabolism of drugs by gut microbiota mainly includes hydrolysis and reduction reactions [17,18,19]. At present, the drug metabolism of gut microbiota is regarded as an important supplement to the body’s drug metabolism [17,20,21,22,23]. Studies have shown that the human gut microbiota is stable and conserved at the phylum level, while there are large differences at the genus and species levels. People with different genetic backgrounds and living habits have great differences in the dominant genus and structural composition of the intestinal flora. For example, *Bacteroides* is the genus with the most relative abundance and the greatest individual differences in Western populations, while *Phascolarctobacterium* is the most abundant genus in Chinese intestinal flora [24,25]. Therefore, affected by individual differences in gut microbiota, there may be differences in the metabolic properties, efficacy, and mechanism of action of natural medicines after oral administration to humans [17]. More and more scholars have begun to incorporate the study of gut microbiota into the research of the in vivo process of drugs, in order to elucidate the mechanism of action of drugs and guide clinical drug use more rationally [26,27,28].

*Saussurea involucrata*, as an oral Chinese medicine, will inevitably interact with gut microbiota after entering the gastrointestinal tract. Therefore, elucidating the interaction between *Saussurea involucrata* and gut microbiota is of great reference value for understanding its pharmacological mechanism in cardio protection, blood lipid regulation, and other aspects. In this study, we established the fingerprint of *Saussurea involucrata* by High Performance Liquid Chromatography (HPLC), and identified four main components of *Saussurea involucrata* by LC/MS^n^-IT-TOF, which were reported to be related to myocardial ischemia protection and cardiovascular protection. At the same time, we used LC-MS/MS and LC/MS^n^-IT-TOF to analyze the metabolism of the main components of *Saussurea involucrata* and the intestinal flora in the co-culture incubation system, and summarized the possible mass spectrometry cleavage pathways. In addition, we also studied the effect of the four main components of *Saussurea involucrata* on the endogenous metabolism (short chain fatty acids) of gut microbiota. This study summarized four main components of *Saussurea involucrata*, a total of five possible main gut microbiota metabolites, and at the same time, for the first time, the bidirectional regulation of *Saussurea involucrata* and intestinal flora was studied. The contributions to myocardial ischemia protection and cardiovascular protection of the bidirectional regulation of *Saussurea involucrata* were also summarized. It is helpful in understanding the metabolism of the main active substances of *Saussurea involucrata* by gut microbiota, and provides new thinking for the basic research of pharmacodynamics and new drug development in cardiovascular protection.

## 2. Results

### 2.1. Four Main Components Were Found in Fingerprint of Saussurea involucrata

In this study, we aimed to determine whether *Saussurea involucrata* could interact with gut microbiota to generate a unique metabolic profile, so as to provide a reference for the unique role of gut microbiota in the efficacy of oral administration of *Saussurea involucrata*.

**Figure 1:**
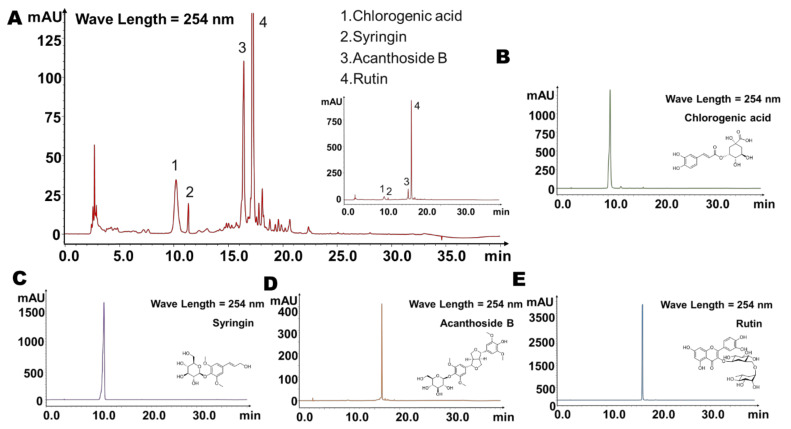
Fingerprint of *Saussurea involucrata*. (**A**) Fingerprint of *Saussurea involucrata*, with the four main components marked in the chromatogram. (**B**) Chromatogram of chlorogenic acid standard. (**C**) Chromatogram of syringin standard. (**D**) Chromatogram of acanthoside B standard. (**E**) Chromatogram of rutin standard.

We first established the fingerprint of the 80% methanol extract of the whole plant of *Saussurea involucrata* by HPLC, and then screened the main components of *Saussurea involucrata*. In the established fingerprints, as shown in Figure 1A, we found that each component was mainly washed out between 5 min and 30 min. In the study of Chinese traditional medicine, the effective substances are more likely to be the largest components. So, we selected four main chromatographic peaks in the fingerprint, which were numbered 1–4, and the corresponding retention times were 10.2 min, 11.3 min, 16.4 min, and 17.3 min, respectively. We used the standard substances for comparison, and under the same analytical conditions, we found that peaks 1–4 corresponded to the retention times of chlorogenic acid, syringin, acanthoside B, and rutin, respectively (Figure 1B–E), suggesting that these four substances may be the main ingredients of *Saussurea involucrata*.

**Figure 2:**
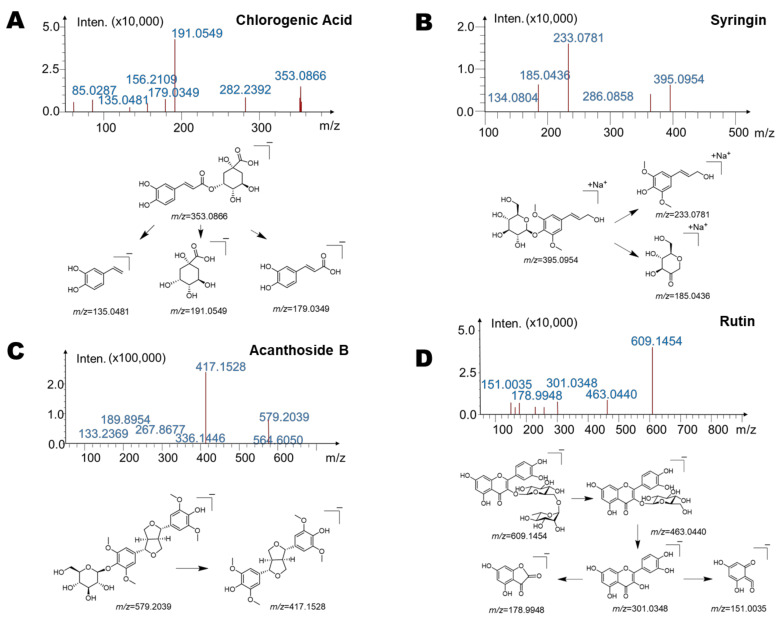
High-resolution mass spectrometry of the four main substances (chlorogenic acid, syringin, acanthoside B, rutin) of *Saussurea involucrata*. (**A**) The mass spectrometric cleavage pathway of chlorogenic acid. (**B**) The mass spectrometric cleavage pathway of syringin. (**C**) The mass spectrometric cleavage pathway of acanthoside B. (**D**) The mass spectrometric cleavage pathway of rutin.

Next, we identified these four main chromatographic peaks using high-resolution mass spectrometry (LC/MS^n^-IT-TOF) (Table 1, Figure 2). The mass spectrum of peak 1 and the fragmentation pathway are shown in Figure 2A. The molecular formula of peak 1 was C_16_H_18_O_9_, with the molecular ion of [M − H]^−^, and *m*/*z* of 353.0866. From the high-resolution mass spectra obtained by IT-TOF, we made the following summary of its mass spectral cleavage pathway. The ion with *m*/*z* of 191.0549 was generated from the cleavage of the ester bond by the molecular ion ([M − H-C_9_H_6_O_3_]^−^, *m*/*z* = 191.0549). The subsequent ion with *m*/*z* of 179.0349 was the product of ions obtained when the molecular ion lost 1 quinicacid group ([M − H-C_7_H_10_O_5_]^−^, *m*/*z* = 179.0349) [29], and the latter continued to lose 1 COO to obtain an ion with *m*/*z* = 135.0481([M − H-C_7_H_10_O_5_-COO]^−^) [29]. Based on peak 1 and its molecular weight and mass spectral fragmentation pathway, and further comparison with the standard substances, we believe that peak 1 was chlorogenic acid [29]. The mass spectrum of peak 2 and the fragmentation pathway are shown in Figure 2B. The molecular formula of peak 2 was C_17_H_24_O_9_, with the molecular ion of [M + Na]^+^, and *m*/*z* of 395.0954 [30]. From the high-resolution mass spectra obtained by IT-TOF, we made the following summary of its mass fragmentation. The ion with *m*/*z* of 233.0781 was generated from the loss of 1 glucose from the molecular ion (M + Na^+^-C_6_H_10_O_5_, *m*/*z* = 233.0781). The subsequent ion with *m*/*z* of 185.0436 was the product of ions produced by the molecular ion cleaving the ether bond and then losing 2H (M + Na^+^-C_11_H_14_O_4_, *m*/*z* = 185.0436). Based on peak 2 and its molecular weight and mass spectral fragmentation pathway, and further comparison with the standard substance, we conjectured that peak 2 was syringin [30]. Next, we analyzed the mass spectrum of peak 3 as well as the fragmentation pathway (Figure 2C). The molecular formula of peak 3 was C_28_H_36_O_13_, and the molecular ion was [M − H]^−^ with *m*/*z* of 579.2039. From the high-resolution mass spectra obtained by IT-TOF, we made the following summary of its mass spectral cleavage pathway. The ion with *m*/*z* of 417.1528 was obtained from the loss of 1 glucose from the molecular ion ([M − H-C_6_H_10_O_5_]^−^, *m*/*z* = 417.1528) [31]. Based on peak 3 and its molecular weight and mass spectral fragmentation pathway, and further comparison with the standard substance, we confirmed that peak 3 was acanthoside B [31]. Finally, we analyzed the mass spectrum of peak 4 as well as the fragmentation pathway (Figure 2D). The molecular formula of peak 4 was C_27_H_30_O_16_, and the molecular ion was [M − H]^−^ with *m*/*z* of 609.1454. From the high-resolution mass spectra obtained by IT-TOF, we make the following summary of its mass fragmentation pathway. The ion with *m*/*z* of 463.0440 was produced by losing 1 rhamnose from the molecular ion ([M − H-C_6_H_10_O_4_]^−^, *m*/*z* = 463.0440) [32]. The subsequent ion with *m*/*z* of 301.0348 was generated from the loss of 1 glucose in the ion of *m*/*z* = 463.0440 ([M − H-C_6_H_10_O_4_-C_6_H_10_O_5_]^−^, *m*/*z* = 301.0348) [33]; the latter continued to lose the B ring on the flavonoid skeleton to generate the ion of [M − H-C_6_H_10_O_4_-C_6_H_10_O_5_-C_7_H_6_O_2_]^−^ with *m*/*z* = 178.9948 [32], and the ion with *m*/*z* = 178.9948 continued to lose the C ring to produce an ion with *m*/*z* = 151.0035 [32]. Based on peak 4 and its molecular weight and mass spectral fragmentation pathway, and further comparison with the standard, we suspected that peak 4 was rutin.

### 2.2. Main Components of Saussurea involucrata Were Significantly Metabolized by the Gut Microbiota

**Figure 3:**
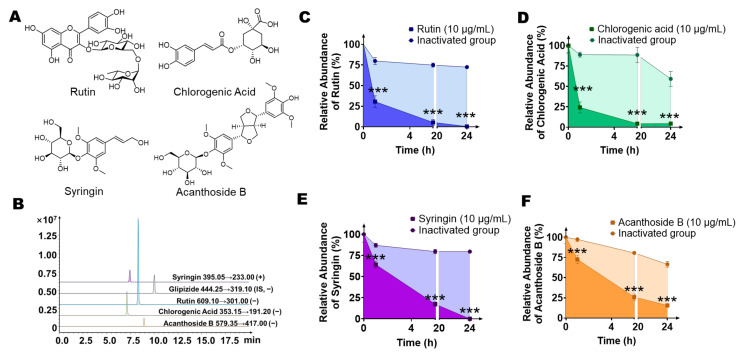
The four main components of *Saussurea involucrata* (chlorogenic acid, syringin, acanthoside B, rutin) were incubated with the gut microbiota incubation system comprising colon contents of seven SD rats and anaerobic medium (weight/volume ratio = 1:20), respectively (the final drug concentration was 10 µg/mL) and the four main components can be metabolized by intestinal bacteria, respectively. (**A**) The chemical structure of the four main components of *Saussurea involucrata*. (**B**) Extracted ion chromatogram (EIC) spectra of syringin, rutin, chlorogenic acid, acanthoside B, and the internal standard (glipizide). (**C**) The level of rutin decreased during incubation with rat intestinal bacteria after 0 h, 1 h, 6 h, and 24 h (*n* = 3). (**D**) The level of chlorogenic acid decreased during incubation with rat intestinal bacteria after 0 h, 1 h, 6 h, and 24 h (*n* = 3). (**E**) The level of syringin decreased during incubation with rat intestinal bacteria after 0 h, 1 h, 6 h, and 24 h (*n* = 3). (**F**) The level of acanthoside B decreased during incubation with rat intestinal bacteria after 0 h, 1 h, 6 h, and 24 h (*n* = 3). Data are presented as mean ± SD, and two-tailed Student’s *t* test was used for analysis (*** *p* < 0.001).

Next, we established a quantitative detection method for the abovementioned four main components (rutin, chlorogenic acid, syringin, acanthoside B) from *Saussurea involucrata* using LC-MS/MS to explore whether the main components of *Saussurea involucrata* can be metabolized by intestinal flora. The molecular structures of the four main components are shown in Figure 3A. In addition, Figure 3B shows the mass chromatogram of the established quantitative detection method; the retention time of rutin in this method was 8.3 min, while that of chlorogenic acid was 7.1 min, that of syringin was 7.4 min, and the retention time of acanthoside B was 8.9 min, while the retention time of the internal standard (glipizide) was 10.0 min.

Then, we incubated the mixture of colonic contents of seven SD rats with rutin, chlorogenic acid, syringin, and acanthoside B (final concentration of 10 μg/mL) for 24 h to explore the effect of gut microbiota on metabolism of the above four components. The incubation system of twice heat-inactivated colon contents was used as a negative control to exclude the interference of environmental factors such as culture medium. Samples were collected at 0 h, 1 h, 6 h, and 24 h after incubation, and the contents of the four prototype components in the system were detected by LC-MS/MS. As shown in Figure 3C, rutin disappeared rapidly in this system. It can be observed that compared with the inactivated negative control, the concentration of the original drug was 69.4%, 94.6%, and 99.5% at 1 h, 6 h, and 24 h, respectively. The inactivated group still maintained 72.4% of the original concentration at 24 h, indicating the strong metabolic effect of intestinal flora on rutin. Next, we explored the metabolic characteristics of chlorogenic acid by gut bacteria. As shown in Figure 3D, chlorogenic acid can be rapidly metabolized by intestinal bacteria in this system. In the first hour, chlorogenic acid was metabolized rapidly. Compared with the starting point, chlorogenic acid was 75.7% metabolized. At 6 h and 24 h, chlorogenic acid was basically completely metabolized, and only 4.3–4.4% remained. However, in the inactivated group, chlorogenic acid maintained a relatively stable concentration in the first 6 h, was still 88.6% at 6 h, and only had a large environmental decomposition effect at 24 h (still 59.2% of the initial concentration), indicating that intestinal bacteria play a key role in the metabolism of oral chlorogenic acid. Next, we further investigated the relationship between syringin metabolism and gut microbiota. As shown in Figure 3E, compared with rutin and chlorogenic acid, syringin was metabolized relatively slowly by intestinal bacteria, only 35.8% was metabolized in the system at 1 h, and the degree of metabolism at 6 h and 24 h was gradually increased, at 82.6% and 99.9% of the original concentration, respectively. The inactivated group still maintained 79.6% of the original concentration at 24 h, indicating that the intestinal flora had a strong metabolic effect on syringin. Finally, we co-incubated acanthoside B with the intestinal bacteria incubation system. Similar to syringin, acanthoside B was metabolized relatively slowly by intestinal bacteria; only 27.3% was metabolized in the system at 1 h, while at 6 h and 24 h, the degree of metabolism gradually increased, with 74.0% and 84.3% of the original concentration metabolized, respectively. The concentration of acanthoside B in the inactivated group was still 66.6% of the original concentration at 24 h, which also indicated that intestinal bacteria played an important role in the metabolism of acanthoside B.

Based on the above results, we found that the main components of *Saussurea involucrata*, rutin, chlorogenic acid, syringin, and acanthoside B, can be metabolized by the intestinal flora, but the contents of the above substances in the inactivated intestinal bacteria system did not change significantly, which indicated that after oral administration of *Saussurea involucrata*, its main components were significantly modified and metabolized by intestinal flora in the gastrointestinal tract, and may produce new or stronger pharmacological activities beyond the original drug.

### 2.3. Identification of Gut Microbiota Metabolites of Main Components (Saussurea involucrata) by HPLC/MS^n^-IT-TOF

In order to explore the main potential metabolites of the four main components in *Saussurea involucrata* in intestinal bacteria, we used LC/MS^n^-IT-TOF to find the suspected metabolites in the metabolic system.

After incubating the main components of *Saussurea involucrata*, rutin, chlorogenic acid, syringin, and acanthoside B with the intestinal bacteria system, we detected a total of five main potential intestinal bacteria metabolites, and the details including retention times, molecular formular, and fragment characteristics are summarized in Table 2. Among them, one major metabolite was detected in the rutin group, which was named M1, and its retention time under the LC/MS^n^-IT-TOF conditions was 19.8 min. Two major metabolites (M2, M3) were detected in the chlorogenic acid group with retention times of 13.1 min and 16.8 min, respectively. At the same time, one major metabolite (M4) was detected in the syringin group with a corresponding retention time of 15.3 min. A major metabolite (M5) was also detected in the acanthoside B group, which appeared at 18.4 min. It is worth noting that the peaks of these metabolites were not detected in the inactivated negative control group, indicating that M1–M5 were not substances produced by environmental effects, but metabolites produced by the metabolism of intestinal flora.

**Figure 4:**
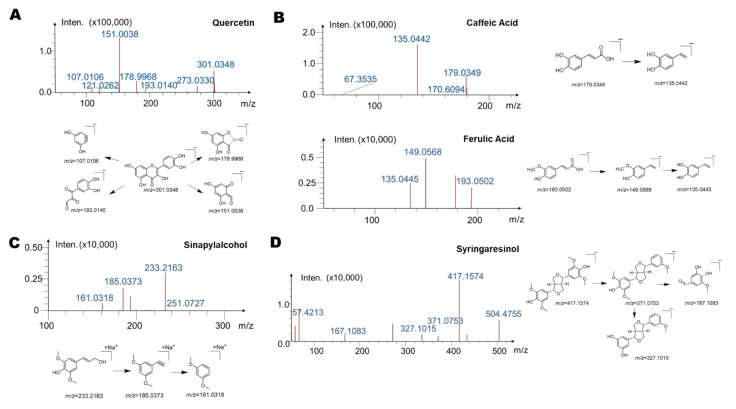
High-resolution mass spectrometry of intestinal bacterial metabolites of four main substances (chlorogenic acid, syringin, acanthoside B, rutin) of *Saussurea involucrata*. (**A**) The mass spectral cleavage pathway of quercetin (metabolite of rutin). (**B**) The mass spectral cleavage pathway of caffeic acid and ferulic acid (metabolites of chlorogenic acid). (**C**) The mass spectral cleavage pathway of sinapylalcohol (metabolite of syringin). (**D**) The mass spectral cleavage pathway of syringaresinol (metabolite of acanthoside B).

Next, we investigated possible structures of abovementioned metabolites. In the rutin group, we summarized the mass spectrum and possible metabolic structure of M1 in Figure 4A. The molecular formula of M1 was C_15_H_10_O_7_; the molecular ion of the metabolite was [M − H]^−^, and its *m*/*z* = 301.0348. From the high-resolution mass spectra obtained by IT-TOF, we made the following summary of its mass spectral cleavage pathway. The ion with *m*/*z* of 178.9968 was obtained from the loss of the B ring of the flavonoid skeleton from the molecular ion [M − H-C_7_H_6_O_2_]^−^, *m*/*z* = 178.9968). The subsequent ion with *m*/*z* of 151.0038 was the product of ions formed by further losing the C ring of the flavonoid skeleton. At the same time, *m*/*z* = 301.0348 can also directly lose the B ring and C ring to produce an ion with *m*/*z* = 107.0106. Finally, the ion with *m*/*z* of 193.0140 was generated after the molecular ion lost the A ring. Based on the molecular structure, molecular weight, and mass spectral cleavage pathway of the prototype drug rutin and M1, and compared with the standard substance, we conjectured that M1 may be the aglycone metabolite of rutin by loss of rutinose, quercetin.

Then, we analyzed the structures of the main metabolites M2 and M3 in the chlorogenic acid group. As shown in Figure 4B, the molecular formula of M2 was C_9_H_8_O_4_, and its molecular ion was [M − H]^−^, with *m*/*z* of 179.0349. From the high-resolution mass spectrum obtained by IT-TOF, we conjectured that the ion with *m*/*z* of 135.0442 was generated from the loss of COO from the molecular ion ([M − H-COO]^−^, *m*/*z* = 135.0442). The molecular formula of M3 was C_10_H_10_O_4_, and its molecular ion was [M − H]^−^, with *m*/*z* of 193.0502. From the high-resolution mass spectrum obtained by IT-TOF, we speculated that the ion with *m*/*z* of 149.0568 was generated from the loss of COO from the molecular ion ([M − H-COO]^−^, *m*/*z* = 149.0568). The latter can further lose CH_2_ to form an ion with *m*/*z* of 135.0442 ([M − H-COO-CH_2_]^−^). Based on the molecular structure, molecular weight, and mass spectral cleavage pathway of the prototype drug chlorogenic acid and M2 and M3, and compared with the standard substance, we confirmed that M2 and M3 may be the aglycone of chlorogenic acid by hydrolyzing glucuronic acid (M2) and the follow-up product after further methylation (M3), respectively. The metabolites of chlorogenic acid were identified as caffeic acid (M2) and ferulic acid (M3).

Further, we summarized the mass spectra of the major metabolite M4 in the syringin group, as well as the possible metabolic structure (Figure 4C). The molecular formula of M4 was C_11_H_14_O_4_, and its molecular ion was [M + Na]^+^, with *m*/*z* of 233.0481. From the high-resolution mass spectra obtained by IT-TOF, we made the following summary of its mass fragmentation pathway. We speculated that the ion with *m*/*z* of 185.0373 was obtained from the loss of CH_4_O_2_ from the molecular ion (M + Na^+^-CH_4_O_2_, *m*/*z* = 185.0373). The ion with *m*/*z* of 161.0318 was generated from the ion with *m*/*z* of 185.0373 by further losing C≡C (M + Na^+^-CH_4_O_2_-C≡C, *m*/*z* = 161.0318). Based on the molecular structure, molecular weight, and mass spectrometry cleavage pathway of the prototype drug syringin and M4, and comparison with the standard substance, we speculated that M4 may be the aglycone metabolite of syringin, sinapylalcohol.

Finally, we performed structural elucidation of the potential major metabolite M5 in the acanthoside B group. As shown in Figure 4D, the molecular formula of M5 was C_22_H_26_O_8_, and its molecular ion was [M − H]^−^, with *m*/*z* of 417.1574. From the high-resolution mass spectra obtained by IT-TOF, we speculated that the ion with *m*/*z* of 371.0753 was produced from the loss of CH_2_O_2_ from the molecular ion ([M − H-CH_2_O_2_]^−^, *m*/*z* = 371.0753). The latter can further lose C_2_H_4_O to obtain an ion with *m*/*z* of 327.1015 ([M − H-CH_2_O_2_-C_2_H_4_O]^−^), and the ion with *m*/*z* of 371.0753 can further split the five-carbon ring and lose CH_2_ to generate the ion with *m*/*z* of 167.1083. Based on the molecular structure, molecular weight, and mass spectral cleavage pathway of the prototype drug acanthoside B and M5, and comparing these with the standard substance, we suspected that M5 may be the aglycone metabolite, syringaresinol.

### 2.4. Quantification of Main Metabolites (Quercetin, Caffeic Acid, Ferulic Acid, Sinapylalcohol, and Syringaresinol) of Gut Microbiota by LC-MS/MS

**Figure 5:**
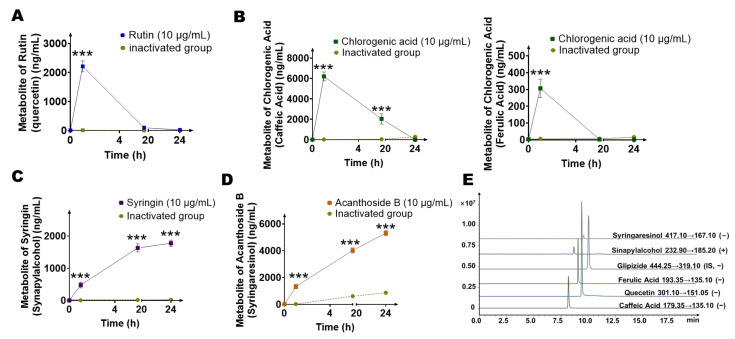
Quantitative analysis of intestinal bacterial metabolites of four main components (chlorogenic acid, syringin, acanthoside B, rutin) of *Saussurea involucrata*. (**A**) Metabolic curve of quercetin (metabolite of rutin) (*n* = 3). (**B**) Metabolic curve of caffeic acid and ferulic acid (metabolites of chlorogenic acid) (*n* = 3). (**C**) Metabolic curve of synapylalcohol (metabolite of syringin) (*n* = 3). (**D**) Metabolic curve of syringaresinol (metabolite of acanthoside B) (*n* = 3). (**E**) Extracted ion chromatogram (EIC) spectra of syringaresinol, sinapylalcohol, ferulic acid, quercetin, caffeic acid, and the internal standard (glipizide). Data are presented as mean ± SD, and two-tailed Student’s *t* test was used for analysis (*** *p* < 0.001).

In order to further quantitatively study the potential metabolites of the four main components of *Saussurea involucrata*, we established a quantitative analysis method for the detection of quercetin, caffeic acid, ferulic acid, sinapylalcohol, and syringaresinol in the intestinal bacterial matrix using LC-MS/MS. The established mass spectrometry chromatogram is shown in Figure 5E. The retention time of quercetin in this method was 9.4 min, the retention time of caffeic acid was 8.2 min, the retention time of ferulic acid was 9.3 min, and the retention time of sinapylalcohol was 8.7 min, while the retention time of syringaresinol was 9.6 min. In addition, the retention time of the internal standard was 10.0 min with this method.

The system composed the gut microbiota and four main components of *Saussurea involucrata* for 24 h, and three times the volume of methanol containing the internal standard was added to the system at 0 h, 1 h, 6 h, and 24 h. Then, we explored the production levels of five potential metabolites in each group, as shown in Figure 5A–D. First, we investigated the production level of quercetin, the hydrolyzed metabolite of rutin. It can be seen from Figure 5A that quercetin was rapidly generated at first, and quercetin reached the highest peak (2210.8 ± 187.0 ng/mL) at 1 h, and then the quercetin concentration gradually decreased, indicating that quercetin was not the final metabolite of rutin, and other reactions may further occur after the metabolite was formed. Next, we investigated the generation of caffeic acid and ferulic acid, the metabolites of chlorogenic acid (Figure 5B). The concentration of caffeic acid reached the highest peak at 1 h (6203.3 ± 433.5 ng/mL), followed by a gradual decrease in caffeic acid concentration. The methylation metabolite of caffeic acid, ferulic acid, had a lower concentration than caffeic acid. It also decreased gradually after 1 h. This showed that neither caffeic acid nor ferulic acid was the final product of metabolite of chlorogenic acid by intestinal bacteria, and other reactions may further occur after the metabolites were generated. Then, we explored the production of sinapylalcohol, the intestinal bacterial metabolite of syringin. As shown in Figure 5C, the concentration of sinapylalcohol increased continuously with the incubation time, and gradually reached a plateau (24 h, 1777.8 ± 94.1 ng/mL) after 6 h (1632.0 ± 125.2 ng/mL). In the acanthoside B group, the concentration of syringaresinol changed with time as shown in Figure 5D. The concentration of syringaresinol increased with the incubation time, and reached the highest concentration (5312.5 ± 196.7 ng/mL) at 24 h, and was still showing an upward trend.

In the inactivated control group corresponding to each group, all metabolites were basically not detected (only the syringaresinol reached 866.7 ng/mL at 24 h, which may be due to environmental factors), indicating that the five main metabolites were mainly produced by the metabolism of gut microbiota.

### 2.5. The Main Components of Saussurea involucrata Induce Short Chain Fatty Acid Production of Gut Microbiota

In order to investigate the influence of the four main components in *Saussurea involucrata* on the endogenous metabolism of intestinal bacteria, we incubated rutin, chlorogenic acid, syringin, and acanthoside B with the intestinal bacteria incubation system for 0, 1, 6, 24 h, and the content of short chain fatty acids in the incubation system was determined to investigate the effects of the four main components in vitro on the production of short chain fatty acids in intestinal bacteria.

**Figure 6:**
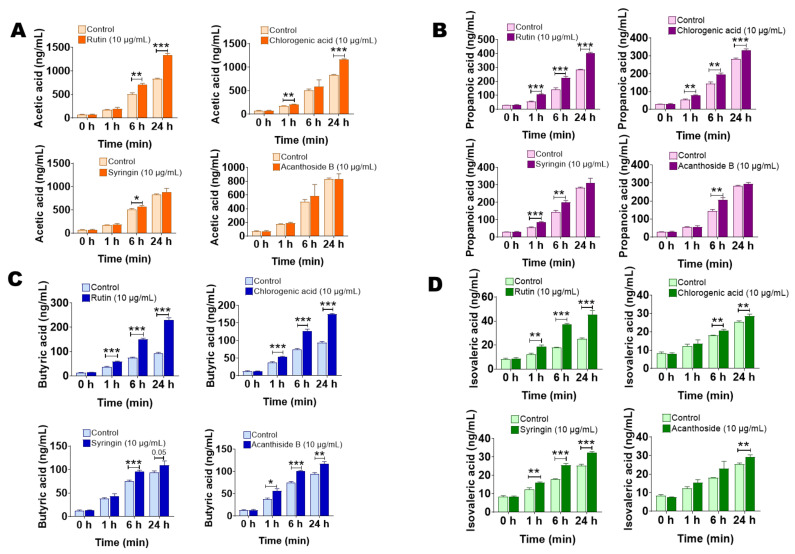
The four main components of *Saussurea involucrata* (chlorogenic acid, syringin, acanthoside B, rutin) stimulated intestinal bacteria to produce short chain fatty acids (*n* = 3). (**A**) Acetic acid; (**B**) propionic acid; (**C**) butyric acid; (**D**) isovaleric acid. Data are presented as mean ± SD, and two-tailed Student’s *t* test was used for analysis (*** *p* < 0.001, ** *p* < 0.01, * *p* < 0.05).

Figure 6A–D shows the effects of the four main components on the production of acetic acid, propionic acid, butyric acid, and isovaleric acid in intestinal bacteria, respectively. As shown in Figure 6A, with the prolongation of incubation time, compared with the control group, it can be found that rutin and chlorogenic acid can significantly increase the content of acetic acid in the incubation system. At 24 h, the acetic acid content in the rutin and chlorogenic acid groups was 1.60 times and 1.40 times that of the control group, respectively. At the same time, syringin group and acanthoside B group had no obvious ability to induce acetic acid. Figure 6B shows the effects of the four main components on the production of propionic acid in intestinal bacteria. Compared with the control group, rutin and chlorogenic acid significantly promoted the production of propionic acid in the incubation system of intestinal bacteria by 41.7% and 16.7%. At the same time, the propionic acid concentration in the syringin group was also increased by 9.5% compared with the control group at 24 h, but there was no obvious difference. However, the acanthoside B group had no obvious ability to induce propionic acid. Next, we tested the butyric acid content in the incubation system. As shown in Figure 6C, compared with the control group, rutin, chlorogenic acid, and acanthoside B could significantly stimulate the synthesis and secretion of butyric acid by intestinal bacteria. At 24 h, the concentration of butyric acid in rutin, chlorogenic acid, and acanthoside B groups increased by 146.3%, 86.4%, and 25.0% respectively, and syringin also promoted the production of butyric acid in the incubation system, but the extent was smaller (16.3% increase at 24 h) and showed no significant difference. Finally, we examined the content of isovaleric acid in each group of incubation systems (Figure 6D). The effects of rutin and syringin on isovaleric acid production were more significant. At 24 h, the content of isovaleric acid in the system increased by 80.0% and 27.9%, respectively, compared with the control group. Chlorogenic acid and acanthoside B also had a certain stimulating effect on isovaleric acid. At 24 h, the content of isovaleric acid in the system increased by 13.1% and 15.9%, respectively.

The above results show that in addition to the significant metabolic effects of intestinal bacteria on the main components of *Saussurea involucrata*, the latter will in turn affect the production and secretion of secondary metabolites of intestinal bacteria, which is very important for the elucidation of the mechanism of oral *Saussurea involucrata* and other natural medicines.

## 3. Discussion

*Saussurea involucrata* is a traditional Chinese medicine commonly used in traditional Chinese patents. It has the functions of warming the kidney and helping *yang*, dispelling wind and eliminating dampness, and promoting blood circulation. In recent years, *Saussurea involucrata* has been reported to have a protective effect against myocardial ischemia [34]. As an oral traditional Chinese medicine, the effective substances of *Saussurea involucrata* may not be limited to the main component itself, and its metabolites should play a very important role. In addition to the influence of the host’s own digestive enzymes, *Saussurea involucrata* orally delivered to the gastrointestinal tract is likely to interact with the gut microbiota. This study mainly focused on the bidirectional regulation of gut microbiota and the main components of *Saussurea involucrata*.

In this article, we first selected four major peaks as the targets in the established fingerprint of *Saussurea involucrata* because in the study of Chinese traditional medicine, the effective substances are more likely to be the largest components. The four components were identified as rutin, chlorogenic acid, syringin, and acanthoside B. Rutin and chlorogenic acid are the quality control components of *Saussurea involucrata* in *Pharmacopoeia of the People’s Republic of China*, and syringin is the quality control component in some Chinese patent medicine containing *Saussurea involucrata*, such as XUE-LIAN oral liquid (XUE-LIAN KOU-FU-YE). So, the four components were selected for further research. The above four substances are reported to have the potential to treat myocardial ischemia. For example, rutin has been reported to significantly reduce serum cGMP and nitric oxide (NO) levels, and inhibit iNOS and 3-nitrotyrosine (3-NT) [13], while showing cardioprotective function in a rat model of ischemia-reperfusion-induced myocardial infarction [12,35]. It has been reported that chlorogenic acid improves the survival rate after myocardial infarction, and it has been proved that chlorogenic acid has a protective effect against myocardial ischemia by reducing the inflammatory response and exerting antioxidant activity [36]. In addition, syringin has been reported to exert cardioprotective effects in diabetic cardiomyopathy through the interaction of TLR4/NF-κB/NLRP3 and PGC1a/SIRT3 pathways [37]. Acanthoside B has shown a dose-dependent nitric oxide inhibitory potential, while restoring the antioxidant status of cells and attenuating inflammatory cytokines [38], which has a potential auxiliary protective effect against myocardial ischemia.

Next, after co-culturing the four components of *Saussurea involucrata* with the gut microbiota incubation system, we found a total of five possible main gut microbiota metabolites. After the molecular weight comparison, they may be hydrolyzed products of glycosidic bonds or methylated products. The molecular formulas are C_15_H_10_O_7_ ([M − H]^−^, *m*/*z* = 301.0348, M1, quercetin, metabolite of rutin), C_9_H_8_O_4_ ([M − H]^−^, *m*/*z* = 179.0349, M2, caffeic acid, metabolite of chlorogenic acid), C_10_H_10_O_4_ ([M − H]^−^, *m*/*z* = 193.0502, M3, ferulic acid, metabolite of chlorogenic acid), C_11_H_14_O_4_ ([M + Na]^+^, *m*/*z* = 233.2163, M4, sinapylalcohol, metabolite of syringin), and C_22_H_26_O_8_ ([M − H]^−^, *m*/*z* = 417.1574, M5, syringaresinol, metabolite of acanthoside B). Their structures and mass spectral fragmentation pathways using ion trap time-of-flight tandem mass spectrometry were summarized. At the same time, we carried out quantitative analysis of metabolites, which was helpful for the study of drug metabolism of intestinal bacteria.

The main metabolites of the rutin, chlorogenic acid, syringin, and acanthoside B found in this study were mainly hydrolyzed products. For example, quercetin was the hydrolyzed product of rutin by loss of rutinose. Caffeic acid was the hydrolyzed product of chlorogenic acid by loss of quinicacid. Sinapylalcohol was the hydrolyzed product of syringin by loss of glucose, and syringaresinol was the hydrolyzed product of acanthoside B by loss of glucose. It has reported that enzymes with hydrolysis activity are widely present in intestinal bacteria. For example, the dominant species in intestinal bacteria, such as *Bifidobacteria* and *Lactobacillus,* have related enzyme systems [39]. For non-monoglycosidic compounds, some genera in intestinal bacteria have evolved into enzymes that specifically hydrolyze these glycosidic bonds, such as *Lactobacillus acidophilus* and other genera that can hydrolyze rhamnosidic bonds by L-rhamnosidase, which can be used for deglycosylation of rutin, isohesperidin, etc. [40,41].

In the literature, there are reports that several possible metabolites may act synergistically with the prototype drug and jointly contribute to the protective effect of *Saussurea involucrata* in myocardial ischemia. For example, quercetin is a hydrolyzed metabolite of rutin. Studies have shown that quercetin, as an *L*-type Ca^2+^ channel inhibitor, has a protective effect against myocardial ischemia by inhibiting Ca^2+^ inflow and myocardial contractility [42]. In addition, quercetin can alleviate the oxidative stress injury of cardiomyocytes by inhibiting NADPH oxidase and xanthine oxidase, blocking the Fenton reaction, and scavenging reactive oxygen species, and at the same time by reducing the response to inflammatory factors and inhibiting apoptosis [43]. Caffeic acid is the hydrolysis metabolite of chlorogenic acid. Caffeic acid can play a cardioprotective role by regulating the activities of cardiac sodium potassium adenosine triphosphate, cardiac magnesium adenosine triphosphate and calcium adenosine triphosphate, affecting cardiac calcium concentration, scavenging free radicals, and improving myocardial cell membrane stability [44]. Ferulic acid, a methylated product of caffeic acid, was reported to alleviate myocardial ischemia-reperfusion injury by upregulating Adenosine 5′-monophosphate (AMP)-activated protein kinase α2 (AMPKα2) expression-mediated ferroptosis inhibition [45]. Sinapylalcohol is a hydrolysis metabolite of syringin, which can reduce the expression levels of iNOS and cyclooxygenase (COX)-2, and effectively inhibit NO, prostaglandin E2 in macrophages (PGE2), and tumor necrosis factor (TNF)-α, thereby exerting anti-inflammatory activity, showing a potential cardioprotective effect [46]. Syringaresinol, the hydrolyzed metabolite of acanthoside B, exerts cardioprotective effects through the Keap1/Nrf2 and TGF-β/Smad signaling pathways, thereby inhibiting inflammation, cardiomyocyte fibrosis, pyroptosis, and oxidative stress [47,48]. It can be seen that the gut microbiota metabolism is likely to be one of the sources of cardiovascular protective pharmacological activity of *Saussurea involucrata*.

Short chain fatty acids refer to saturated fatty acids with backbones containing less than six carbons [49]. Intestinal bacteria can produce short chain fatty acids by breaking down carbohydrates, branched-chain amino acids, fats, and other substances in the gastrointestinal tract. Since intestinal bacteria are rich in metabolic enzymes that the host does not have, such as propionyl-CoA transferase, propionaldehyde dehydrogenase, etc., the short chain fatty acids in the body are mainly generated by intestinal bacteria [50,51]. In our study, the main components of *Saussurea involucrata* induced and stimulated intestinal bacteria to produce short chain fatty acids, including acetate, propionate, butyrate, and isovaleric acid. There are three main ways that exogenous substances affect the production of short chain fatty acids by gut microbiota: (1) Directly promoting or inhibiting the growth of specific intestinal bacteria, some exogenous molecules themselves can destroy the integrity of the bacterial membrane or inhibit the activity of enzymes, thereby inhibiting bacterial growth. In addition, some exogenous molecules can directly act as prebiotics to promote the growth of certain bacterial cells or improve the activity of related enzymes, such as rutin and chlorogenic acid [52,53]; (2) Indirectly promoting or inhibiting the growth of certain species of bacteria, such as inhibiting the colonization of pathogenic bacteria by competitive exclusion and inducing host immune responses [54]. (3) Exogenous substances serve as carbon sources to provide precursors of short chain fatty acids, which can be used by intestinal bacteria as the only carbon source [55], and the glycosyl moiety of glycosides is eventually metabolized into short chain fatty acids by intestinal bacteria. Furthermore, our results showed that the induction of butyric acid was the most significant by each component. It has been reported that butyrate acid content and the abundance of butyrate-producing bacteria are significantly negatively correlated with cardiovascular disease. For example, Zhu et al. [56] reported that the abundance of *Escherichia-Shigella* and *Enterococcus* increased in patients with atherosclerosis and heart failure, and the abundance of butyrate producers, *Faecalibacterium*, *Roseburia,* and *Eubacterium rectale* decreased. Jie et al. [57] reported that a reduction in the overall genetic potential for butyrate production could be observed in metagenomic data from patients with atherosclerosis. Butyrate can regulate energy expenditure and induce mitochondrial function through the function of its cellular receptors, free fatty acid receptor 2 (FFAR2) or free fatty acid receptor 3 (FFAR3) [58], thus it can be involved in inflammation, glucagon-like peptide-1 (GLP-1) secretion and body energy regulation [59,60,61,62], which in turn play a role in the prevention of obesity, atherosclerosis, and other cardiovascular diseases. Other short chain fatty acids, such as acetic acid, have been shown to reduce cardiac fibrosis and improve cardiac function in mouse models [63]. In addition, propionic acid has also shown similar biological activity to butyric acid after entering the blood [64].

Of course, in this study, we also observed that the concentration of some intestinal bacteria metabolites first increased and then decreased in the incubation system, such as quercetin, etc., indicating that these substances may not be the final intestinal bacteria metabolites. It has been reported that the A-ring part of the flavonoid skeleton of quercetin first generates polyphenols, such as phloroglucinol [65,66,67]. After undergoing enol interconversion, aromaticity of phloroglucinol is greatly reduced, and it is further decomposed by some intestinal bacteria with the assistance of NAD(P)H, and finally generates short chain fatty acids, which can be further used for bacterial ATP synthesis. In addition, after the metabolites of gut microbiota are absorbed into the body, they can further react with the liver enzymes in the human body, which further expands the therapeutic effect. For example, quercetin enters the brain through the systemic circulation and accumulates in the brain in the form of quercetin-*3*-*O*-glucuronic acid, which can improve brain neuroinflammation [68,69]. This also indirectly illustrates the importance of study of intestinal flora metabolism of *Saussurea involucrata*.

Of course, this study also has certain limitations. For example, the fingerprint of the *Saussurea involucrata* can be affected by the origin of the plant, the processing of the drug, etc. Multiple origins of the drug can be involved for further study. Colon contents of SD rats were used to explore the metabolic characteristics of gut microbiota, and the composition and distribution of the intestinal bacteria among different species are different, which may lead to dissimilar results. Therefore, validation with different species is needed to further expand the implications of this experiment. The low-level active components of *Saussurea involucrata* are also worthy of exploration, which requires more sensitive analysis methods and more in-depth metabolic research, and with the study of cardiovascular diseases and gut microbiota [70,71,72,73,74], more targets and mechanism of *Saussurea involucrata* will be found.

**Figure 7:**
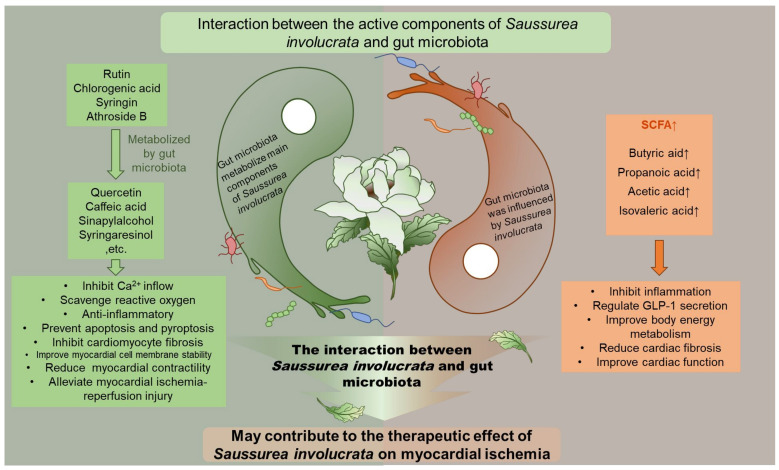
The gut microbiota interacts with the four main components of *Saussurea involucrata* (chlorogenic acid, syringin, acanthoside B, rutin). First, gut bacteria can metabolize the four main components of *Saussurea involucrata* (chlorogenic acid, syringin, acanthoside B, rutin) into the corresponding intestinal bacteria metabolites, which have potential therapeutic effects against myocardial ischemia; in addition, the four main components of *Saussurea involucrata* (chlorogenic acid, syringin, acanthoside B, rutin) can also in turn stimulate the production of short chain fatty acids (acetic acid, propionic acid, butyric acid, isovaleric acid) by intestinal bacteria, which further contributes to the protective effect of *Saussurea involucrata* against myocardial ischemia.

In summary, this study focused on the bidirectional regulation of gut microbiota and the main components of *Saussurea involucrata*. Fingerprints using HPLC were established and the main components with myocardial ischemia-protective activity were selected. LC-MS/MS and LC/MS^n^-IT-TOF were carried out for structural identification and quantitative studies on intestinal bacterial metabolites. The effect of the main components of *Saussurea involucrata* on the endogenous metabolism of intestinal bacteria such as short chain fatty acid production was also studied. These potential mechanisms are summarized in Figure 7, which helps to understand the effective substance of the main components of *Saussurea involucrata* in myocardial ischemia protection and gives guidance for future clinical use.

## 4. Materials and Methods

### 4.1. Instruments and Reagents

Rutin (CAS, 153-18-4), chlorogenic acid (CAS, 327-97-9), syringin (CAS, 118-34-3), quercetin (CAS, 117-39-5), caffeic acid (CAS, 331-39-5), ferulic acid (CAS, 1135-24-6), and glipizide (CAS, 29094-61-9) were purchased from Solarbio Life Sciences Co., Ltd. (Beijing, China). Acanthoside B (CAS, 7374-79-0), sinapylalcohol (CAS, 537-33-7), and syringaresinol (CAS, 1177-14-6) were purchased from Shanghaiyuanye Bio-Technology Co., Ltd., Shanghai, China. The purity of all standard reagents was qualified for quantitative analysis. HPLC grade methanol, HPLC grade acetonitrile, HPLC grade acetic acid, and LC/MS grade formic acid were purchased from Fisher Scientific (Fair Lawn, NJ, USA). The High-Performance Liquid Chromatograph (HPLC) was purchased from Shimadzu Corporation (Kyoto, Japan), and coupled with a triple quadrupole mass spectrometer from Shimadzu Corporation (Kyoto, Japan), LCMS-8060, used for the quantitative detection. Another HPLC tandem quadrupole time-of-flight mass spectrometer from Shimadzu Corporation (Kyoto, Japan), LC-MSn-IT-TOF, was applied for the qualitative identification and structural analysis of metabolites. A shaking incubator was purchased from Longyue Instrument Co., Ltd. (Shanghai, China). A WH-681 vortex mixer was purchased from Jintan Shenglan Instrument Manufacturing Co., Ltd., Jintan, China. The refrigerated high-speed centrifuge was purchased from Eppendorf (Hamburg, Germany).

### 4.2. Animals

Seven Sprague Dawley adult male rats (200–300 g) were purchased from SPF (Beijing, China) Biotechnology Co., Ltd. All animals had free access to food and water, and were housed in a ventilated room with a circulation of 12 h of light and 12 h of darkness. The temperature was maintained at 20–24 °C and humidity 40–60%. The rats were fasted for 12 h and allowed to drink freely before the experiment. This study was approved by the Experimental Animal Ethics Committee of the Chinese Academy of Medical Sciences and Peking Union Medical College (No. 00003539, date of approval, 10 April 2022), and strictly followed the instruction of Organizational Guidelines and Ethics Guidelines of the Experimental Animal Ethics Committee.

### 4.3. Fingerprints of Saussurea involucrata by HPLC and LC-MS^n^-IT-TOF

Half a gram of *Saussurea involucrata* powder (which was passed through a 40-mesh sieve) was accurately weighed for a total of 6 portions. Then, 50 mL of 80% methanol was added to each portion, and the sample was extracted ultrasonically for 1 h. Then the sample extract was centrifuged at 4000× *g* for 10 min and passed through a 0.20 μm organic phase syringe filter. The continuous filtrate was used for chromatographic analysis. The analytical conditions were as follows:

An Alltima column (250 × 4.6 mm, 5 μm, GRACE, Chicago, IL, USA) was used for component separation. The flow rate was 0.4 mL/min, and the column temperature was 40 °C. The injection volume was 20 μL. Mobile phase: 0.2% acetic acid/water (mobile phase A) and methanol (mobile phase B). Gradient elution (B%): 0.01 min, 20% → 10.00 min, 30% → 15.00 min, 60% → 25.00 min, 80% → 30.00 min, 80% → 40.00 min, stop. The autosampler temperature was set to 4 °C. The detection wavelength for PDA was 254 nm.

The quantitative detection of main components of *Saussurea involucrata* was conducted by comparing the standard substances and the fingerprints with LC/MS^n^-IT-TOF. The liquid chromatography method was the same as mentioned above. The mass spectrometry conditions were set as follows: CDL temperature, 160 °C; heating block temperature, 200 °C; nebulizing gas flow rate, 1.5 L/min; detector voltage, 1.76 kV; and collision energy, 70%. Automatic detection mode was used for fragmentation, with a primary *m*/*z* ratio ranging from 50 to 800 and a secondary *m*/*z* ratio ranging from 50 to 800.

### 4.4. Determination of Four Main Components of Saussurea involucrata by LC-MS/MS

The quantitative detection of four main components of *Saussurea involucrata* was performed using LCMS-8060 equipped with an ESI ion source. An Agilent ZORBAX Eclipse XDB-C8 column (4.6 μm × 150 mm, 5 μm, Agilent, Santa Clara, CA, USA) was applied for separation of the analytes. The flow rate was 0.4 mL/min, and the temperature of column oven was 40 °C. The injection volume was 5 μL. The mobile phase used was 0.1% acetic acid: water as mobile phase A and acetonitrile as mobile phase B. Gradient elution conditions (B%) were as follows: 0.01 min, 10% → 2.00 min, 12% → 5.00 min, 28% → 7.50 min, 95% → 11.00 min, 95% → 16.00 min, 10% → 20.00 min, stop. The MRM mode was used for detection by the mass spectrometer, with mass transitions for rutin (MRM−) of 609.10 → 301.00 (Q1 pre bias: 18.0 V, CE: 25.0 V, Q3 pre bias: 18.0 V, dwell time: 50 ms), chlorogenic acid (MRM−) of 353.15 → 191.20 (Q1 pre bias: 10.0 V, CE: 22.0 V, Q3 pre bias: 11.0 V, dwell time: 50 ms), syringin (MRM+) of 395.05 → 233.00 (Q1 pre bias: −13.0 V, CE: −26.0 V, Q3 pre bias: −17.0 V, dwell time: 50 ms), acanthoside B (MRM−) of 579.35 → 417.00 (Q1 pre bias: 18.0 V, CE: 25.0 V, Q3 pre bias: 24.0 V, dwell time: 50 ms), and the internal standard (IS, glipizide) of 444.25 → 319.10 (MRM−, Q1 pre bias: 18.0 V, CE: 18.0 V, Q3 pre bias: 24.0 V, dwell time: 50 ms), respectively. The mass spectrometer parameters were set as follows: nebulizer gas, 3.0 L/min; heating gas, 10 L/min; interface temperature, 300 °C; DL temperature, 250 °C; heat block temperature, 400 °C; drying gas, 10 L/min; interface voltage, −4.5 kV; and CID gas pressure, 270 kPa.

The quantitative detection of possible metabolites of the four main components of *Saussurea involucrata* was performed using LCMS-8060 equipped with an ESI ion source. An Agilent ZORBAX Eclipse XDB-C8 column (4.6 μm × 150 mm, 5 μm, Agilent, Santa Clara, CA, USA) was applied for separation of the analytes. The flow rate was 0.4 mL/min, and the temperature of column oven was 40 °C. The injection volume was 5 μL. The mobile phase used was 0.1% acetic acid: water as mobile phase A and acetonitrile as mobile phase B. Gradient elution condition (B%) was as follows: 0.01 min, 10% → 2.00 min, 12% → 5.00 min, 28% → 7.50 min, 95% → 11.00 min, 95% → 16.00 min, 10% → 20.00 min, stop. The MRM mode was used for detection by the mass spectrometer, with mass transitions for quercetin (MRM−) of 301.10 → 351.05 (Q1 pre bias: 23.0 V, CE: 27.0 V, Q3 pre bias: 18.0 V, dwell time: 50 ms), caffeic acid (MRM−) of 179.35 → 135.10 (Q1 pre bias: 12.0 V, CE: 23.0 V, Q3 pre bias: 11.0 V, dwell time: 50 ms), ferulic acid (MRM−) of 193.35 → 135.10 (Q1 pre bias: 14.0 V, CE: 21.0 V, Q3 pre bias: 10.0 V, dwell time: 50 ms), sinapylalcohol (MRM+) of 232.90 → 185.20 (Q1 pre bias: −23.0 V, CE: −39.0 V, Q3 pre bias: −27.0 V, dwell time: 50 ms), syringaresinol (MRM−) of 417.10 → 167.10 (Q1 pre bias: 28.0 V, CE: 42.0 V, Q3 pre bias: 35.0 V, dwell time: 50 ms), and the internal standard (IS, glipizide) of 444.25 → 319.10 (MRM−, Q1 pre bias: 18.0 V, CE: 18.0 V, Q3 pre bias: 24.0 V, dwell time: 50 ms), respectively. The mass spectrometer parameters were set as follows: nebulizer gas, 3.0 L/min; heating gas, 10 L/min; interface temperature, 300 °C; DL temperature, 250 °C; heat block temperature, 400 °C; drying gas, 10 L/min; interface voltage, −4.5 kV; and CID gas pressure, 270 kPa.

All the samples were maintained at 4 °C before injection.

### 4.5. Identification of the Metabolites (Main Components of Saussurea involucrata) by LC/MS^n^-IT-TOF

The qualitative identification of possible metabolites (main components of *Saussurea involucrata*) was performed using LC/MS^n^-IT-TOF equipped with an ESI ion source. An Alltima column (250 × 4.6 mm, 5 μm, GRACE, Chicago, IL, USA) was used for component separation. The flow rate was 0.4 mL/min, and the column temperature was 40 °C. The injection volume was 20 μL. Mobile phase: 0.2% acetic acid/water (mobile phase A) and methanol (mobile phase B). Gradient elution (B%): 0.01 min, 20% → 10.00 min, 30% → 15.00 min, 60% → 25.00 min, 80% → 30.00 min, 80% → 40.00 min, stop. The autosampler temperature was set to 4 °C. The detection wavelength for PDA was 254 nm. The mass spectrometry conditions were set as follows: CDL temperature, 160 °C; heating block temperature, 200 °C; nebulizing gas flow rate, 1.5 L/min; detector voltage, 1.76 kV; and collision energy, 70%. Automatic detection mode was used for fragmentation, with a primary *m*/*z* ratio ranging from 50 to 800 and a secondary *m*/*z* ratio ranging from 50 to 800.

### 4.6. Determination of Short Chain Fatty Acid In Vitro

The Gas Chromatograph (GC-2020) was purchased from Shimadzu Corporation (Kyoto, Japan). Quantification of short chain fatty acid by GC was performed as described previously [59]. A high-polarity Alltech capillary column (AT-WAX, 30 m × 0.25 mm × 0.25 μm, Alltech Cooperation, Chicago, IL, USA) was used for separation with parameters as follows: nitrogen flow, 1.27 mL/min; purge flow, 3.0 mL/min; total pressure, 105.0 kPa; injection port, 230 °C; and FID detector, 250 °C, respectively. The temperature programming started with 80 °C for 1 min, and linearly increased to 130 °C at the rate of 5 °C/min, and maintained for 5 min.

### 4.7. In Vitro Incubation of Four Main Components of Saussurea involucrata with Gut Microbiota

After 7 SD rats were sacrificed, the colon contents were collected and added to the sterilized anaerobic medium (Solarbio Life Sciences Co., Ltd., Beijing, China) at a ratio of 1.0 g: 20 mL, and stirred gently. After filtering, the culture medium containing gut microbiota (mixed medium) was placed in a N_2_ atmosphere, and pre-incubated at 37 °C for 60 min before use. We accurately weighed 1.0 mg of rutin, chlorogenic acid, syringin, and acanthoside B, respectively. Then, dissolved them with methanol to obtain solutions of 1 mg/mL, respectively. The incubation system consisted of 10 μL of rutin (or the other 3 solutions respectively) in methanol (1 mg/mL), and 990 μL of mixed medium. The incubation was conducted in a 37 °C, 200 rpm shaking incubator. The incubation system was maintained in a completely anaerobic environment during the experiment. The solution and mixed medium were incubated for 0, 1, 6, and 24 h, respectively. In addition, the negative control group was introduced consisting of twice-boiled mixed medium incubated with the same amount of solutions.

For qualification and quantification of the main components and possible metabolites by LCMS-8060 and LC/MS^n^-IT-TOF, the termination reaction was carried out by adding 3-fold volume of 100 ng/mL glipizide methanol solution (IS), shaking it evenly and then precipitating the protein. After each sample was centrifuged at 13,400 rpm in a 4 °C refrigerated centrifuge for 10 min, 5 μL of supernatant was injected for LC-MS/MS analysis, and 20 μL of supernatant was injected for LC/MS^n^-IT-TOF analysis.

Then, 1 mg/mL of rutin, 1 mg/mL of chlorogenic acid, 1 mg/mL of syringin and 1 mg/mL of acanthoside B methanol solution was gradually diluted to a series of stock solutions with concentrations of 200 μg/mL, 100 μg/mL, 20 μg/mL, 10 μg/mL, 2 μg/mL, 1 μg/mL, 0.2 μg/mL, and 0.1 μg/mL, respectively. Standard samples were composed of a series of 10 μL of stock solutions and 990 μL of inactivated medium, for quantification of the metabolism of 4 main components of Saussurea involucrata. We prepared a mixed solution containing 1 mg/mL of quercetin, 1 mg/mL of caffeic acid, 1 mg/mL of ferulic acid, 1 mg/mL of sinapylalcohol, and 1 mg/mL of syringaresinol, respectively, and gradually added methanol to obtain a series of mixed stock solutions of 200 μg/mL, 100 μg/mL, 20 μg/mL, 10 μg/mL, 2 μg/mL, 1 μg/mL, 0.2 μg/mL, and 0.1 μg/mL. Mixed standard samples were composed of a series of 10 μL mixed stock solutions and 990 μL inactivated medium, for quantification of potential metabolites of the 4 main components. The rest of the sample processing steps were the same as above.

For detection of short chain fatty acids, at the end of the incubation reaction, acetone (with 1% phosphoric acid, *v/v*) for short chain fatty acid extraction was added. The mixture was centrifuged at 14,000 rpm for 10 min at 4 °C and the supernatant was directly injected into GC for analysis as mentioned before [75].

### 4.8. Statistical Analysis

Mass spectrum data acquisition and subsequent data processing were performed with Shimadzu LabSolutions (Kyoto, Japan) for analysis of GC, HPLC, LCMS-8060 and LC/MS^n^-IT-TOF. Two-tailed ANOVA and Student’s *t*-test were used for statistical analysis with GraphPad Prism Version 5 (GraphPad Software, San Diego, CA, USA). Data are expressed as the mean ± standard deviation (SD), and *p* values less than 0.05 were considered statistically significant.

## Figures and Tables

**Table 1:** Characteristics of main components of *Saussurea involucrata* by LC/MS^n^-IT-TOF.

Components	Retention Time (Min)	Predicted Molecular Weight	Molecular Formula	Fragment Characteristics	
				MS	MS/MS
Chlorogenic acid	10.2	354.31	C_16_H_18_O_9_	353 [M − H]^−^	135, 191, 179
Syringin	11.3	372.37	C_17_H_24_O_9_	395 [M + Na]^+^	233, 185
Acanthoside B	16.4	580.59	C_28_H_36_O_13_	579 [M − H]^−^	417
Rutin	17.3	610.52	C_27_H_30_O_16_	609 [M − H]^−^	463, 301, 178, 151

**Table 2:** Characteristics of main metabolites of main components (*Saussurea involucrata*) in gut microbiota by LC/MS^n^-IT-TOF.

Components	Retention Time (Min)	Reaction	Predicted Molecular Weight	Molecular Formula	Fragment Characteristics	
					MS	MS/MS
M1	19.8	—Rutinose	302.24	C_15_H_10_O_7_	301 [M − H]^−^	193, 178, 151, 107
M2	13.1	—Glucuronic Acid	180.15	C_9_H_8_O_4_	179 [M − H]^−^	135
M3	16.8	—Glucuronic Acid, Methylation	194.18	C_10_H_10_O_4_	193 [M − H]^−^	149, 135
M4	15.3	—Glucose	210.23	C_11_H_14_O_4_	233 [M + Na]^+^	185, 161
M5	18.4	—Glucose	418.44	C_22_H_26_O_8_	417 [M − H]^−^	371, 327, 167

## Data Availability

The data in this study are available in this article.
